# *Klebsiella* virus UPM2146 lyses multiple drug-resistant *Klebsiella pneumoniae in vitro* and *in vivo*

**DOI:** 10.1371/journal.pone.0245354

**Published:** 2021-01-08

**Authors:** Omar Assafiri, Adelene Ai-Lian Song, Geok Hun Tan, Irwan Hanish, Amalia Mohd Hashim, Khatijah Yusoff

**Affiliations:** 1 Faculty of Biotechnology and Biomolecular Sciences, Department of Microbiology, Universiti Putra Malaysia, Serdang, Selangor, Malaysia; 2 Faculty of Agriculture, Department of Agriculture Technology, Universiti Putra Malaysia, Serdang, Selangor, Malaysia; 3 Institute of Biosciences, Universiti Putra Malaysia, Serdang, Selangor, Malaysia; 4 Halal Products Research Institute, Universiti Putra Malaysia, Serdang, Selangor, Malaysia; Cornell University, UNITED STATES

## Abstract

*Klebsiella pneumoniae* are opportunistic bacteria found in the gut. In recent years they have been associated with nosocomial infections. The increased incidence of multiple drug-resistant *K*. *pneumoniae* makes it necessary to find new alternatives to treat the disease. In this study, phage UPM2146 was isolated from a polluted lake which can lyse its host *K*. *pneumoniae* ATCC BAA-2146. Observation from TEM shows that UPM2146 belongs to *Caudoviriales* (Order) based on morphological appearance. Whole genome analysis of UPM2146 showed that its genome comprises 160,795 bp encoding for 214 putative open reading frames (ORFs). Phylogenetic analysis revealed that the phage belongs to *Ackermannviridae* (Family) under the *Caudoviriales*. UPM2146 produces clear plaques with high titers of 10^10^ PFU/ml. The phage has an adsorption period of 4 min, latent period of 20 min, rise period of 5 min, and releases approximately 20 PFU/ bacteria at Multiplicity of Infection (MOI) of 0.001. UPM2146 has a narrow host-range and can lyse 5 out of 22 *K*. *pneumoniae* isolates (22.72%) based on spot test and efficiency of plating (EOP). The zebrafish larvae model was used to test the efficacy of UPM2146 in lysing its host. Based on colony forming unit counts, UPM2146 was able to completely lyse its host at 10 hours onwards. Moreover, we show that the phage is safe to be used in the treatment against *K*. *pneumoniae* infections in the zebrafish model.

## Introduction

*Klebsiella pneumoniae* is a Gram-negative bacterium which can colonize the gastrointestinal tract, lungs, and leads to liver abscesses, pneumoniae, and bacteremia in human [1]. It belongs to the ESKAPE group (*Enterococcus faecium*, *Staphylococcus aureus*, *K*. *pneumoniae*, *Acinetobacter baumannii*, *Pseudomonas aeruginosa*, *and Enterobacter* species) which is responsible for many nosocomial infections in hospitals [2]. The number of these drug-resistant bacteria is on the rise due to the spread of plasmids expressing the β-lactamase enzyme which renders antibiotics that has lactam enzyme as a functional group useless (NDM-1) [3]. Furthermore, the number of new antibiotic groups being discovered has dwindled down drastically compared to the rates in which bacteria gain resistance towards the antibiotics [4]. Thus, it is necessary to search for an alternative to treatments using antibiotics such as phage therapy. For instance, phage cocktail (Kp152, Kp154, Kp155, Kp164, Kp6377, and HD001) with an inactivated sulfamethoxazole-trimethoprim was able to protect a patient from *K*. *pneumoniae* Urinary Tract Infection (UTI) [5].

Bacteriophages are highly selective in infecting and lysing their bacterial hosts [6]. In addition, they exhibit far fewer side effects compared to the antibiotics [7]. At least 70 *K*. *pneumoniae* phages have been isolated and characterized to date [8]. However, very few *K*. *pneumoniae* phages have been studied *in vivo* using mouse, rat and pig models [9–13]. Apart from these animals, the virulence of *K*. *pneumoniae* has also been evaluated in other non-conventional models such as amoeba and zebrafish to mitigate limitations in the mouse, rat and pig models such as high cost, specialized facilities and ethical concerns [8]. It has been shown that only certain *K*. *pneumoniae* strains were able to infect zebrafish and cause mortality. In another study, it was shown that a bioactive molecule from *Streptomyces* sp. was able to reduce mortality of zebrafish infected with *K*. *pneumoniae* [14]. However, there are no publications on phage therapy against *K*. *pneumoniae* using the zebrafish larvae model although this has been studied for other pathogens such as *Vibrio anguillarum* [15] and *Enterococcus faecalis* [16]. In the present study, UPM2146 was isolated, characterized, and tested for its efficacy against *K*. *pneumoniae* 2146 *in vivo* using a zebrafish larvae model.

## Material and methods

### Bacterial strains and experimental conditions

Multiple drug-resistant *Klebsiella pneumoniae* ATCC BAA-2146 was used as the host for UPM2146. *K*. *pneumoniae* IMR-1 (non-drug resistant strain), multiple drug-resistant ATCC BAA-1705 and clinical isolates A01 until A18, and A20 [17], multi-drug resistant *Staphylococcus aureus* 10, and multi-drug resistant *Escherichia coli* ATCC 25922 and *E*. *coli* Top 10 (non-drug resistant) were also used in this study. Dr Erin Lim of Perdana University generously gifted *K*. *pneumoniae* IMR-1, ATCC BAA-2146, ATCC BAA-1705 and *Escherichia coli* ATCC 25922. Clinical isolates *K*. *pneumoniae* A01-A018 and A20 were gifted by Dr Siti Norbaya of Faculty of Medicine and Health Sciences, Universiti Putra Malaysia. The following experimental condition has been applied for all bacteria throughout the experiments unless stated otherwise. A purified single colony was taken from LB agar plate and re-grown overnight in LB broth with shaking at 37°C. On the next day, the bacteria were grown to log phase [5 × 10^8^ colony-forming unit (CFU)/ml] at an OD_600nm_ = 0.6.

### Isolation, enrichment and purification of phage

UPM2146 was isolated from a polluted lake using *K*. *pneumoniae* 2146 as host. Approximately 50 ml of the lake water was centrifuged for 10 min at 6000 × g, 4°C. The supernatant was filtered using a 0.45 μm membrane filter. In order to enrich the phages, 100 μl of *K*. *pneumoniae* 2146 (OD_600nm_ = 0.6) in 0.1 M CaCl_2_ were added to 10 ml of the filtered supernatant before being made up to 30 ml with 5 × LB broth (final concentration of 3.33× LB) (Merck, Germany) and shaken overnight at 37°C. The mixture was centrifuged for 10 min at 8000 × g at 4°C. The supernatant was filtered again. The presence of phage was confirmed through spotting onto a soft overlay agar containing the bacteria. Phages resulting in a positive spot test were then subjected to dilution and plated using the double-layer agar method [18]. Single plaques formed on plates were cut from the double layer agar and placed back into SM buffer [100 mM NaCl, 8 mM MgSO_4_· 7 H_2_O, 50 mM Tris-Cl (1 M, pH 7.5), and 0.01 g of gelatin)] overnight at 4°C, filtered and subjected to double-layer agar again the next day. The same process was repeated three times to ensure purified phages were obtained which showed uniformed distribution of plaques per plate. High-titered phage was prepared from plates flooded with SM buffer, which was centrifuged and filtered. The filtrate was precipitated using 1.5 mM of NaCl and 12% of PEG-8000 to phage lysate and left at 4°C overnight. On the next day, the lysate was centrifuged and the pellet was resuspended in 1 ml of sterile SM buffer with 1 ml of 1 M KCl. After being left on ice for 1 h, the mixture was re-centrifuged at the same speed and temperature. The supernatant which consisted of purified concentrated phage was stored at 4°C.

### Adsorption rate of isolated phages

Determination of the adsorption rate of phage was performed according to Hyman & Abedon [19]. Mid log phase host bacteria (OD_600nm_ = 0.6) were infected with a (1 × 10^10^ PFU/ml) of UPM2146 at MOI of 0.01 at 37°C. After infection, the phage titers at every 2 min for 10 min were determined. The samples (30 ml) were centrifuged at 5 000 × g for 3 min at 4°C to remove the bacteria and adsorbed phage. The concentration of free phage was determined by double-layer agar method.

Adsorption rate of phage was calculated as:

$$\frac{\text{Initial}\;\text{titer}\;\text{of}\;\text{phage-titer}\;\text{of}\;\text{free}\;\text{phage}}{\text{Initial}\;\text{titer}\;\text{of}\;\text{phage}}\;\times \;100\%$$

### Host range and efficiency of plating (EOP)

The host range of UPM2146 was determined according to D’Andrea *et al*. [20]. The phage was tested against multiple sub-species of *K*. *pneumoniae* (IMR-1 non-drug resistant strain, ATCC BAA-2146, ATCC BAA-1705, A01 until A18, and A20), multi-drug resistant *Staphylococcus aureus* 10, and *Escherichia coli* (ATCC 25922 and Top 10). Ten μl of the phage were spotted onto an overlay agar containing the bacterial host. The agar plates were incubated overnight at 37°C. EOP of bacteria strains which were positive for the spot test was determined against *K*. *pneumoniae* 2146. One hundred μl of UPM2146 were mixed with 100 μl of bacteria, followed by double-layer assay for plaque count at MOI of 1. The EOP of UPM2146 was calculated by dividing the phage titers on the target bacterium (*K*. *pneumoniae* 2146, A5, A7, A13, and IMR-1) by the phage titers on their original isolated host (*K*. *pneumoniae* 2146). EOP was classified as ‘High’ when the ratio of target to original host was at least 50% (EOP≥0.5), ‘Medium’ when the EOP was in between 10% to 50% (0.1 ≤EOP<0.5), ‘Low’ when less than 10%, and ‘Inefficient” when EOP was less than 0.1% (EOP≤0.001) [21].

### One-step growth curve

One-step growth experiment was performed according to Kutter & Sulakvelidze [22] to determine the latency periods and burst sizes of UPM2146. Briefly, *K*. *pneumoniae* 2146 was grown to a concentration of 1 × 10^10^ CFU/ml and mixed with phage lysate UPM2146 at MOI = 0.001 before incubation in a shaker at 37°C and 180 rpm. Aliquots of the mixture were then assayed using the double-layer agar method at every 5 min for 30 min.

### The stability of the phage

The stability of the phage at different pH and temperatures was determined according to Taj *et al*. [23] with some modifications. For thermal stability, UPM2146 (10^10^ PFU/ml) was incubated at 4°C, 14°C, 24°C, 37°C, 45°C, 60°C, and 70°C for 1 h in SM buffer and then the phage titer was measured. For pH stability, UPM2146 (10^10^ PFU/ml) was incubated at a pH of 2–14 for 1 h at 37°C in LB broth before the titer was measured using the double-layer agar overlay method. Statistical analysis was performed using SPSS v.23 Chicago: SPSS Inc. One-Way ANOVA was performed followed by post-hoc multiple comparison using Tukey HSD test to determine differences in the phage titer between different treatments. Statistical significance was assumed when p<0.05.

### Turbidity assay

The effectiveness of the phage in clearing its host was tested through turbidity assay. Fifty μl of the phage was mixed with *K*. *pneumoniae* 2146 at log phase at different MOI of 0.02, 0.2, and 2 and incubated by shaking at 37°C, 180 rpm. The OD_600 nm_ was measured at every 30 min. At the peak of the growth curve of *K*. *pneumoniae* which was at OD_600nm_ 0.8 for this analysis, a plate count was also conducted to determine the total reduction of *K*. *pneumoniae* in CFU [24].

### Transmission electron microscopy

Sample preparation was done via 38% sucrose gradient for cushioning in Beckman Coulter Class S ultracentrifuge using SW 40 Ti rotor at a speed of 288 000 × g for 4 h at 4°C [25]. Then, thin carbon support films were prepared by sublimation of a carbon thread onto a freshly cleaved mica surface. Phage was adsorbed onto the carbon film and negatively stained with 2% (w/v) aqueous uranyl acetate, pH 5.0. The sample was visualized using JEM-2100F field emission electron microscope, 200 kV FE (Field Emission). Morphological appearance of the phage was determined based on Goodridge *et al*. [26] with some modifications. Capsid diameter, tail length, and width were analyzed from electron micrographs with ImageJ software Ver1.48 (Rasband, Ver 1.48).

### DNA sequencing and bioinformatics analysis of the phage genome

The DNA was isolated using modified conventional method [27]. The purity and concentration of the phage DNA were determined using Nanophotometer (Implen GmbH, Germany) [28]. Next-generation sequencing was performed on UPM2146 using Illumina Hiseq sequencing by Shanghai Biozeron Biotechnology (PE150 mode), which provided 100× sequence coverage. Then, raw sequencing data were generated by Illumina base-calling software CASAVA v1.8.2 (http://support.illumina.com/sequencing/sequencing_software/casava.ilmn). Adaptor or primer sequences were trimmed using Trimmomatic (http://www.usadellab.org/cms/uploads/supplementary/Trimmomatic) with default parameters. Low quality reads (Q<20) were filtered using the same software. The Sequence’s quality was checked using FastQC. The clean reads were assembled with multiple-Kmer parameters using ABySS (http://www.bcgsc.ca/platform/bioinfo/software/abyss) [29]. GapCloser software (https://sourceforge.net/projects/soapdenovo2/files/GapCloser/) was used to close the gaps between contigs and correct the single base polymorphism for the final assembly results. Gene structural and functional annotation was performed through the RAST (Rapid Annotation using Subsystem Technology) [30]. Determination of lytic or lysogenic lifecycle was predicted using PHACTS [31]. Phylogenetic tree of UPM2146 was constructed to determine the evolutionary relationship of UPM2146 with other existing phages using FASTME. The evolutionary intergenomic distance was estimated using Genome BLAST Distance Phylogeny (GBDP) method [32] and used to infer a balanced minimum evolution tree with branch support via FASTME, including SPR postprocessing [33] for formula D6 respectively. Branch support was inferred from 100 pseudo-bootstrap replicates each. Trees were rooted at the midpoint [34] and visualized with FigTree [35]. A phylogenetic tree based on the amino acid sequence of UPM2146 putative peptidoglycan-binding protein was also constructed using Molecular Evolutionary Genetics Analysis Version X (MEGA X) [36]. Multiple sequence alignment was conducted using ClustalW using default parameters, followed by manual correction of the sequence alignment where necessary. Initial tree(s) for the heuristic search were obtained automatically by applying Maximum Likelihood and BioNJ algorithms to a matrix of pairwise distances estimated using a JTT model, and then selecting the topology with superior log likelihood value. Pairwise distance was estimated using Poisson correction model. All nucleotide sequences of the phage genome and peptidoglycan-binding protein used as reference sequences to create the dendrograms were obtained from Genbank (https://www.ncbi.nlm.nih.gov/nucleotide). GenBank accession number for each of the reference genomes and genes were denoted in the trees.

Comparison between UPM2146 with *Klebsiella* virus 0507KN21 was visualized using Easyfig. All pairwise comparisons of the nucleotide sequences were conducted using the Genome-BLAST Distance Phylogeny (GBDP) method [37] under settings recommended for prokaryotic viruses [38].

Taxon boundaries at the species, genus and family level were estimated with the OPTSIL program [39], the recommended clustering thresholds [38] and an F value (fraction of links required for cluster fusion) of 0.5 [40].

### Effect of UPM2146 on *K*. *pneumoniae* using zebrafish larvae

Immersion assays for *K*. *pneumoniae* were adapted from similar methods performed previously to study *S*. *typhimurium* virulence [41]. Bacterial cultures were grown until exponential phase (OD_600nm_ = 0.4–0.6) and the cells were serially diluted until 10^6^ CFU/ml. Zebrafish larvae (Danio Assay Laboratories Sdn. Bhd., Malaysia) (3 days-post-fertilization) were washed with DANIO media and groups of 10 larvae were placed per well in sterile 6-well plates for the following treatments: (1) Larvae infected with *K*. *pneumoniae* 2146 (3.3 × 10^6^ CFU/ml) (2) Larvae infected with *K*. *pneumoniae* 2146 (3.3 × 10^6^ CFU/ml) and treated with UPM2146 (1 × 10^6^ PFU/ml) (MOI ≈ 0.3), (3) Untreated larvae (DANIO buffer with LB broth) as the negative control, (4) Untreated larvae (DANIO buffer with LB broth and SM buffer) as the negative control. The zebrafish larvae were incubated into two sets at the following times, 30 min and 90 min respectively of each of the group followed by two times washing before adding them into new 6-well plates filled with DANIO buffer, then phage (1 × 10^6^ PFU/ml) was added directly into the new 6-well plate for Group 2. Each group were performed in triplicates. To evaluate the CFU count of *K*. *pneumoniae* 2146 in the infected larvae, 10 larvae from each group were sampled at an interval of 2 h in each of the groups, euthanized via 0.015 M tricaine (pH 7); thus immobilizing the zebrafish larvae. The bacterium on the surface of the larvae were washed away by 2 × washings in DANIO buffer. The larvae were homogenized with 100 μl using Triton X100, followed by adding 12.5 μl of Kanamycin (30 mg/ml) and then the homogenate was spread plated onto Simmons Citrate agar (Merck) before being incubated overnight at 37°C.

## Results

### Morphological characterization and nomenclature of the phage

**Fig 1:**
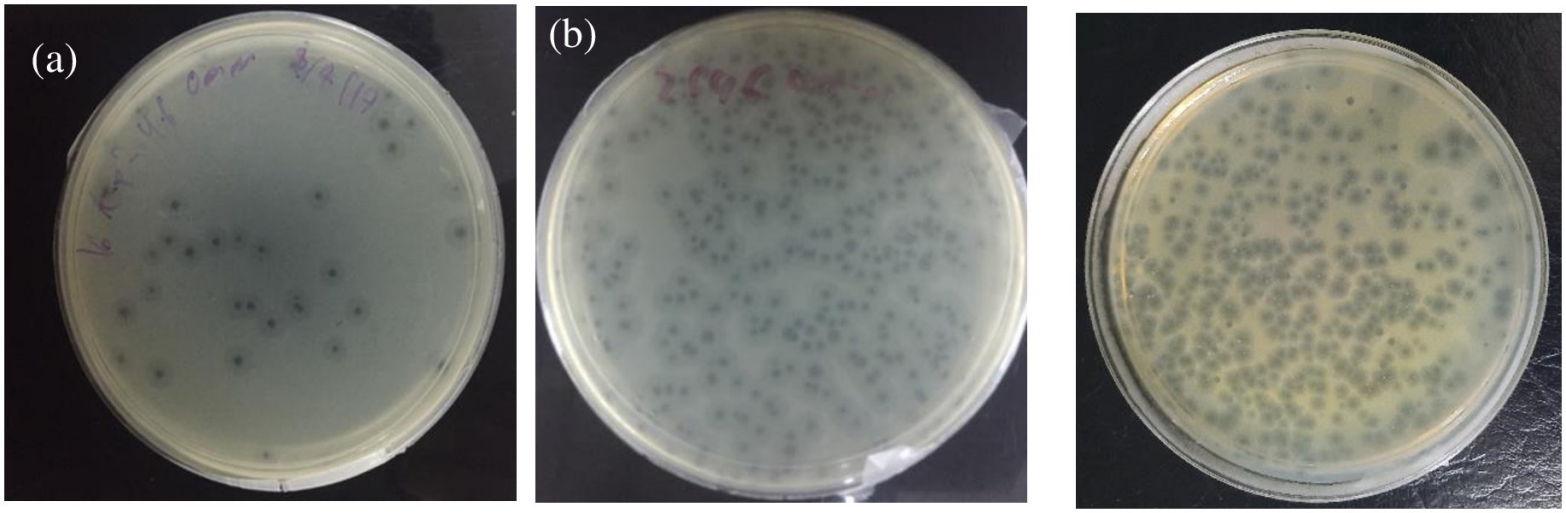
(a) Plaque morphology of UPM2146 was uniformly distributed after overnight incubation at 37°C. (b) The maximum size of plaques ranged from 0.7–1 cm with halo zone of 0.5 cm forming around it, at 36 hours incubation, 37°C.

**Fig 2:**
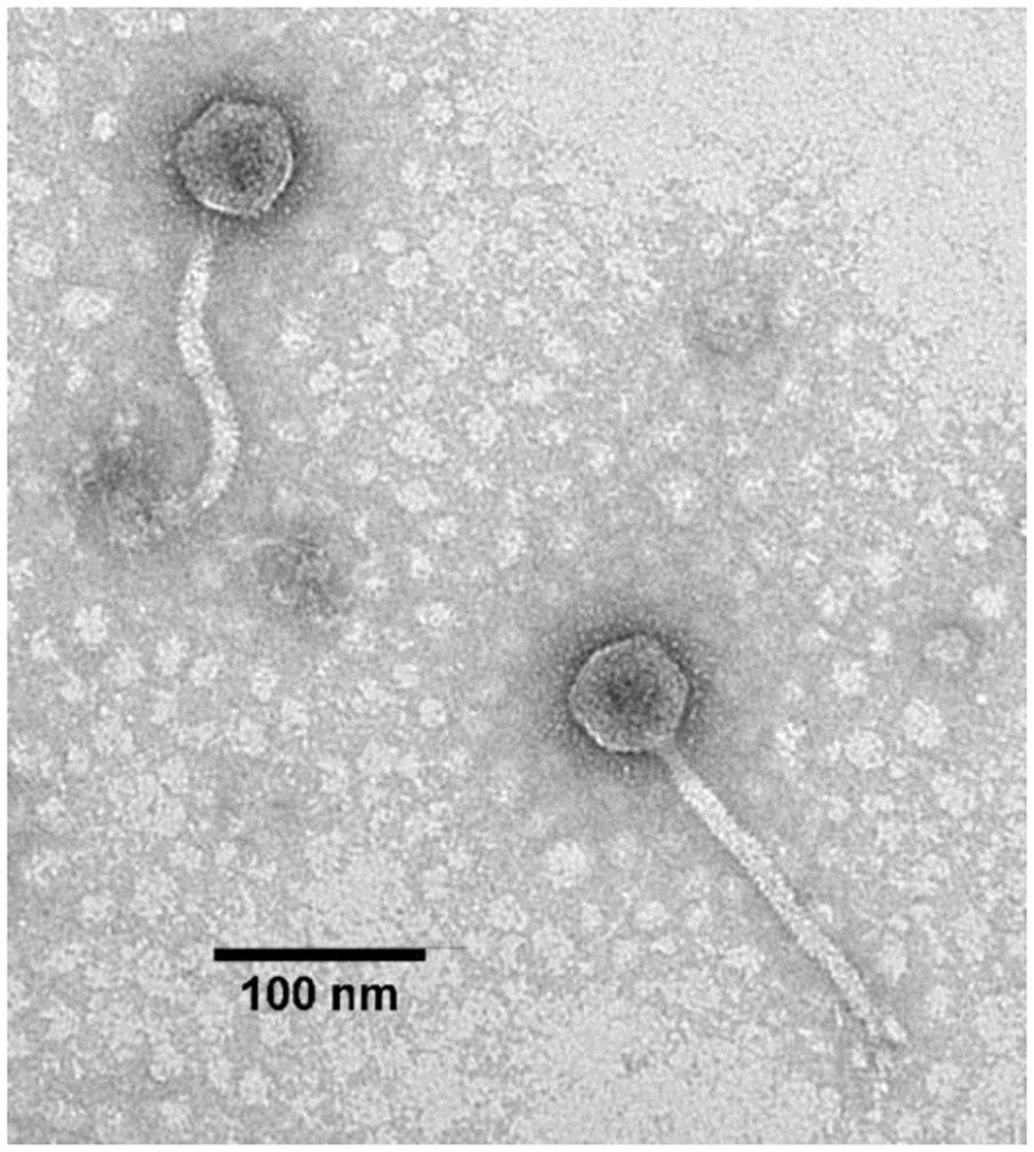
TEM micrograph of UPM2146, indicating that it belongs to *Caudovirales* order. 100 nm scaled bar is shown.

Phage UPM2146 was isolated from a polluted lake in Serdang, Malaysia. It formed clear uniform plaques after overnight incubation at 37°C (Fig 1a), and the maximum size of plaques ranged between 0.7–1 cm in diameter on *K*. *pneumoniae* 2146 with a halo zone (0.5 cm) forming around the plaque after 36 h of incubation at 37°C (Fig 1b). Transmission electron microscopy showed that UPM2146 had an icosahedral head of 51.3 ± 1.8 nm, with a long non- contractile flexible tail of 173.4 ± 1.9 nm in length and a 10.8 ± 7.7 nm in width. Moreover, the presence of the neck, but no collar was observed (Fig 2). Based on these observations, UPM2146 belongs to *Caudovirales* order, according to ICTV [42] (Fig 2).

### Host range and efficiency of plating (EOP) for UPM2146

UPM2146 was tested against a panel of *K*. *pneumoniae* that includes ATCC BAA-2146, ATCC BAA-1705, clinical isolates A01 until A18, and A20. Besides *K*. *pneumoniae*, it was also tested on multi-drug resistant *Staphylococcus aureus* 10, and *Escherichia coli* strains ATCC 25922 and Top 10 using spot test assay. The EOP was used to distinguish the discrepancies that arise between spot test assay and double-layer agar method [21]. Using the spot test, UPM2146 was able to lyse 5 out of 22 *K*. *pneumoniae* tested (22.7%) and did not lyse any of the other bacteria genera. However, based on the results as summarized in Table 1, only the EOP of UPM2146 against its original host which was *K*. *pneumoniae* 2146 showed High EOP while the other four strains which were positive on spot test showed either Low or Inefficient EOP.

**Table 1:** Phage lytic activity against bacterial strains used in this study.

Bacteria	Spot Test (%)	High (EOP≥0.5)	Medium (EOP>0.1<0.5)	Low (EOP≤0.1)	No Activity (EOP<0.001)
*Klebsiella pneumoniae* Clinical isolate (n = 19)	3 (15.59%)	0	0	1	2
*Klebsiella pneumoniae* ATTC strain (n = 2)	1 (50%)	1	0	0	0
*Klebsiella pneumoniae* non-drug resistant strain (n = 1)	1 (100%)	0	0	1	0
Multi-drug resistance *Staphylococcus aureus* clinical isolate (n = 1)	0 (0%)	0	0	0	0
*Escherichia coli* ATTC strain (n = 1)	0 (0%)	0	0	0	0
*Escherichia coli* non-drug resistant strain (n = 1)	0 (0%)	0	0	0	0

EOP was calculated using double-layer method. EOP is classified as ‘High’ only when the ratio of compared bacterium to their host is at minimum or higher than 50% (EOP≥0.5), ‘Medium’ when the EOP is in between 10% and 50% (0.1≤EOP<0.5), ‘Low’ when less than 10%, and ‘Inefficient’ when EOP is less than 0.1% (EOP≤0.001).

### Phage characterization: One-step growth curve, the biological activity of the phage, and turbidity assay

**Fig 3:**
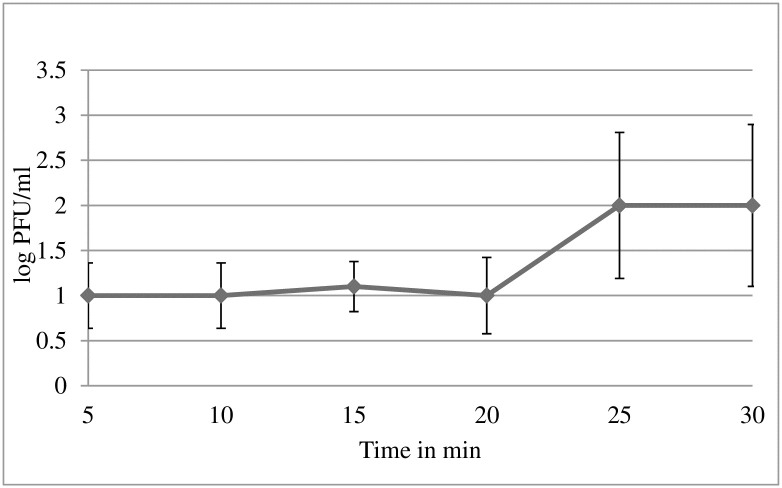
Growth curve of phage UPM2146. One step growth curve of UPM2146 taking 20 min of latent period, 5 min of rise period, and exponential phase of 10 min. Total time required to lyse its host was 25 min that is characteristic of lytic phages. The experiment was done in triplicates.

**Fig 4:**
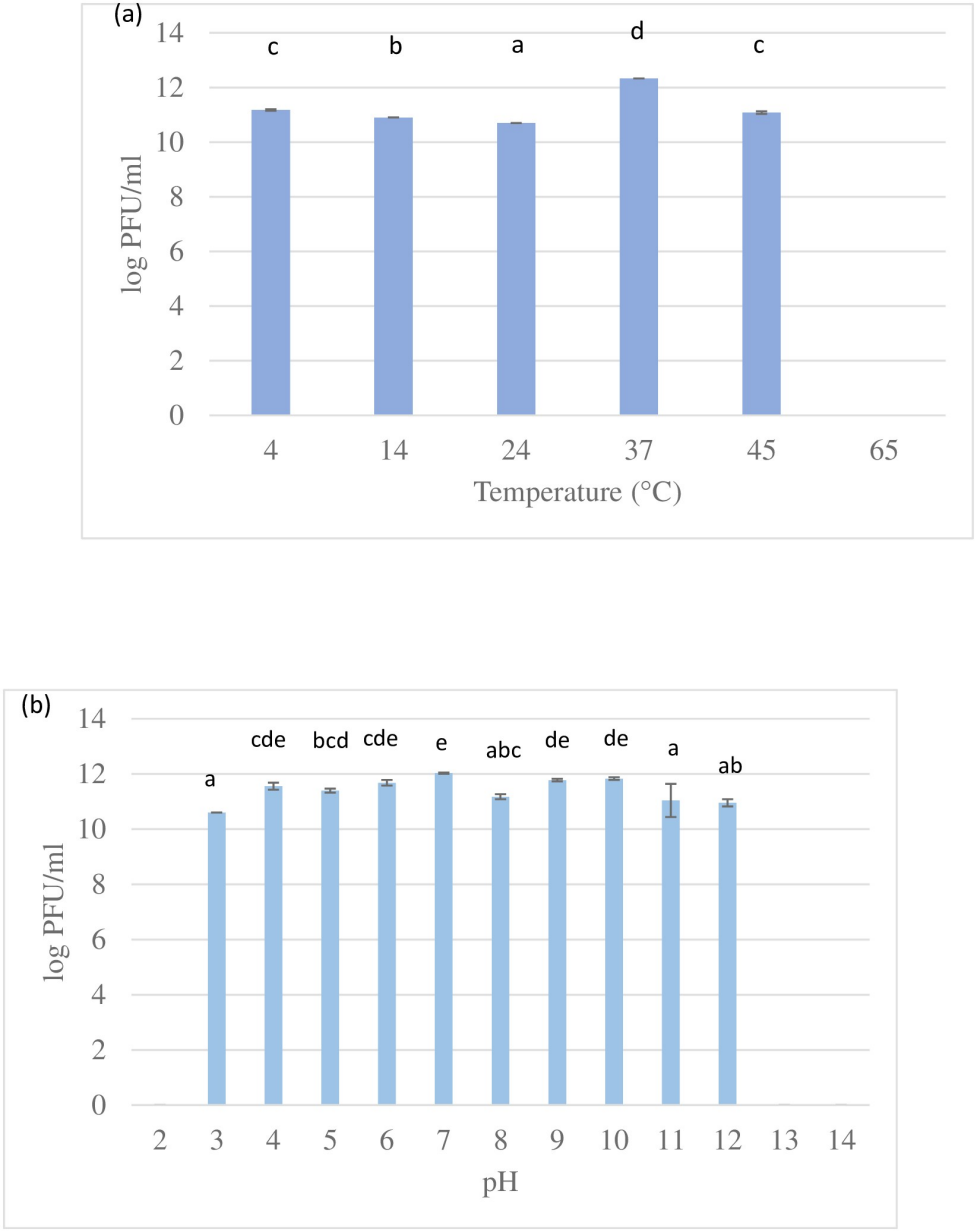
(a) UPM 2146 optimum temperature was at 37°C but was stable at all temperatures tested except at 65 °C and (b) UPM 2146 was stable from pH 3 to 12; thus indicating the potential for it to be used in phage therapy. Bars with different letters indicate significant difference at p<0.05 (Tukey HSD post-hoc test). Error bars are standard deviations (n = 3).

**Fig 5:**
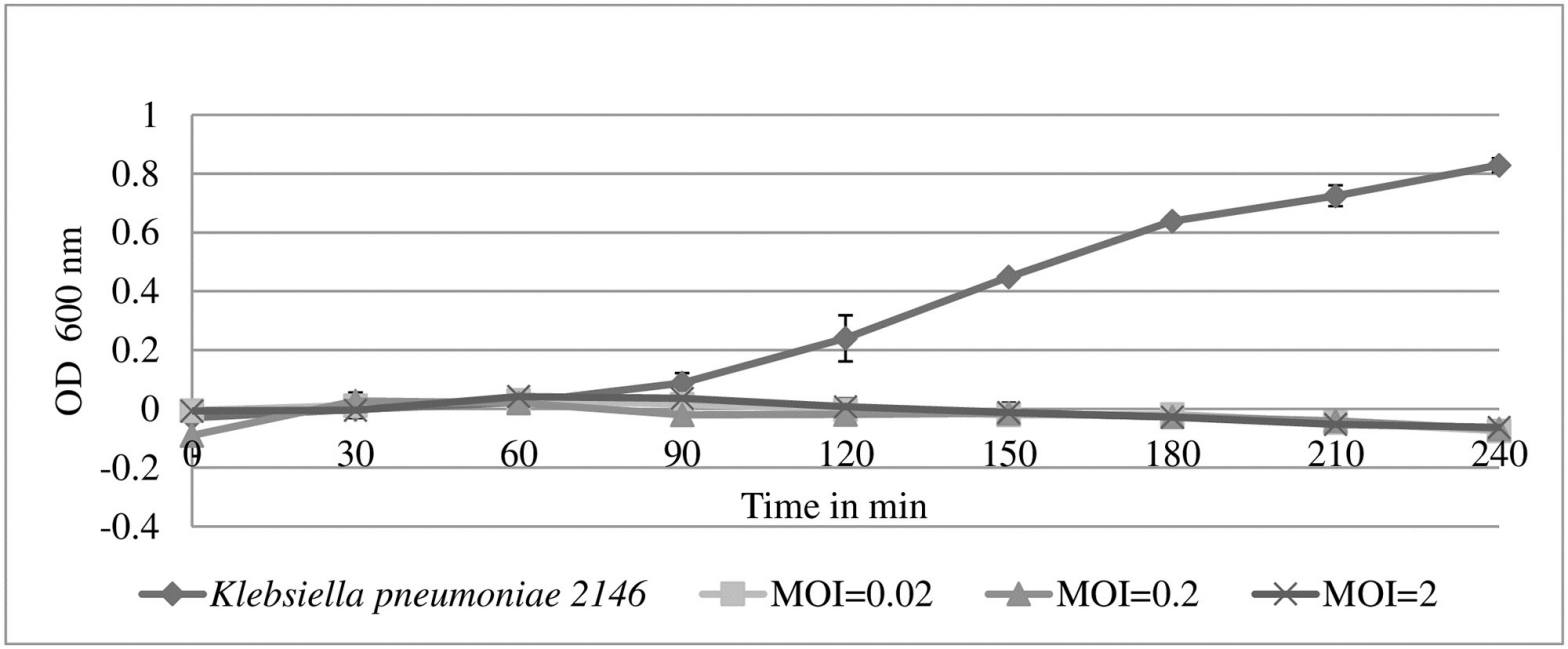
Turbidity assay of UPM21646 against *K*. *pneumonia* 2146. UPM2146 was able to lyse its host and clear the turbidity at 60 min for MOI of 0.02, 0.2 and 2. The bacterial count of control was 1.5±0.104 × 10^9^ CFU/ml at an OD_600nm_ of 0.8. The experiment was done in triplicates.

Adsorption is the first step in a phage life cycle that includes attachment of phage to the surface of *K*. *pneumoniae* 2146. In the present study, 100% of UPM2146 was able to adhere onto the surface of its host bacteria between 2 to 10 minutes. An MOI of 0.001 was used for the one-step growth curve experiment. UPM2146 had a burst size of 20 PFU/bacteria cell, adsorption period of 5 minutes, a latent period of 20 min, and a rise period of 5 min (Fig 3). UPM2146 was stable at all temperatures tested except that it lost its lytic activity at 65°C.and within all pHs tested except the extreme pH of 2, 13, and 14 (Fig 4a & 4b). This also indicates that UPM2146 has the capability to survive at temperature of 37°C and pH 7 making it suitable for therapy purposes. The efficacy of the phage in clearing the bacteria was measured using turbidity assay at OD_600nm_. After adding UPM2146 to *K*. *pneumoniae* 2146 host, the optical density at 600 nm was measured for different MOIs (0.02, 0.2 and 2). At the peak of the *K*. *pneumoniae* growth after 240 min and without phage treatment, the OD_600nm_ was at 0.8 which was equivalent to the bacterial count of 1.5±0.104 × 10^9^ CFU/ml. With phage treatment at all tested MOIs; the bacterial count was reduced to zero. UPM2146 was able to completely lyse the host at 60 min for all different MOIs (Fig 5).

### Genomic analysis and annotation

**Fig 6:**
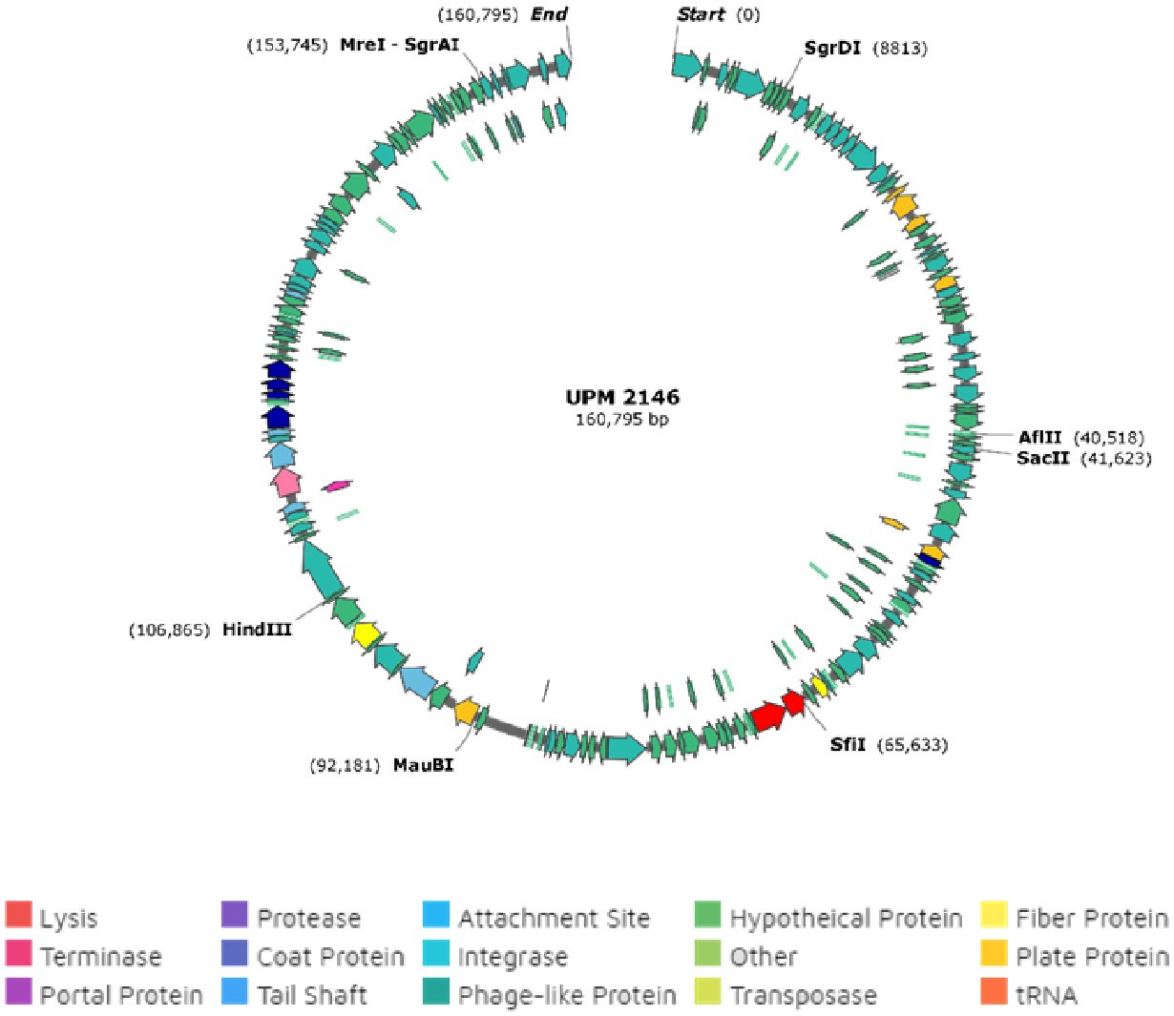
Whole genomic sequence of UPM2146 showing the absence of lysogenic genes such as integrase and toxin genes. This indicates the potential of UPM2416 in phage therapy. The circular genome map was constructed using SnapGene.

**Fig 7:**
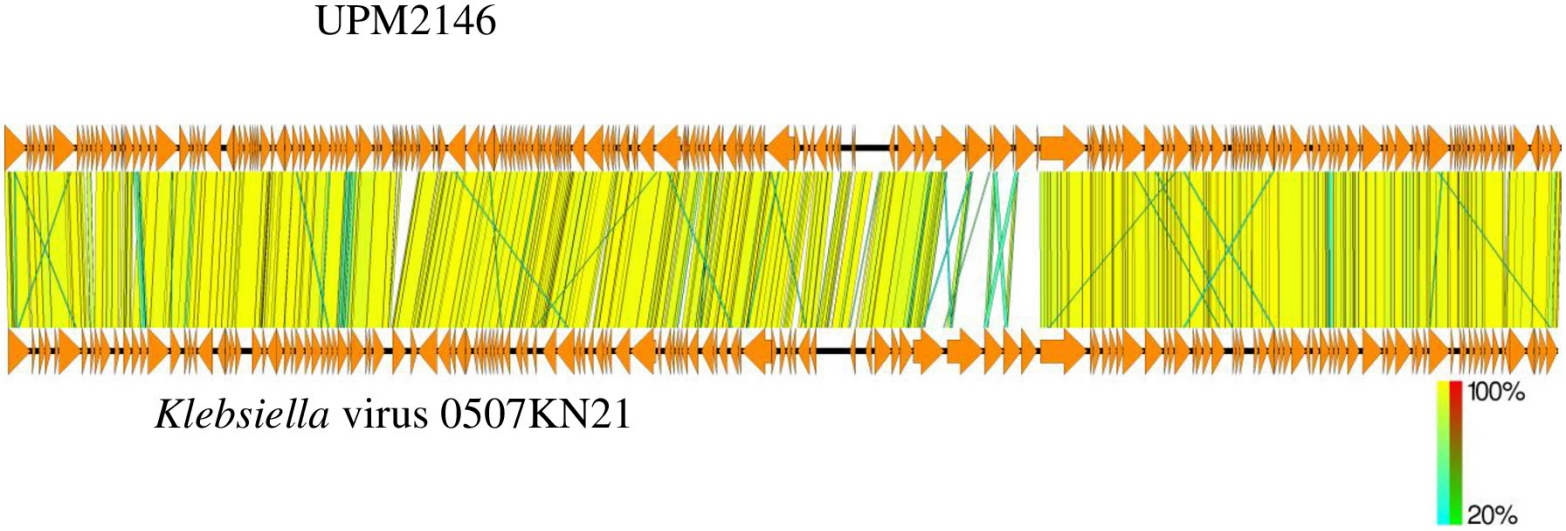
Genomic comparison between UPM2146 and *Klebsiella* virus 0507KN21 analysed using Easyfig. Genomes of each phage is represented by linear visualization of their coding sequences (CDS) represented by arrows. The direction of the arrows indicates the direction of transcription of each CDS. The black vertical lines between the two genomic sequences indicate regions of shared similarities (89%) according to BLASTn. The intensity of these lines indicates the degree of similarity. This confirms that UPM2146 belongs to *Ackermannviridae*.

**Fig 8:**
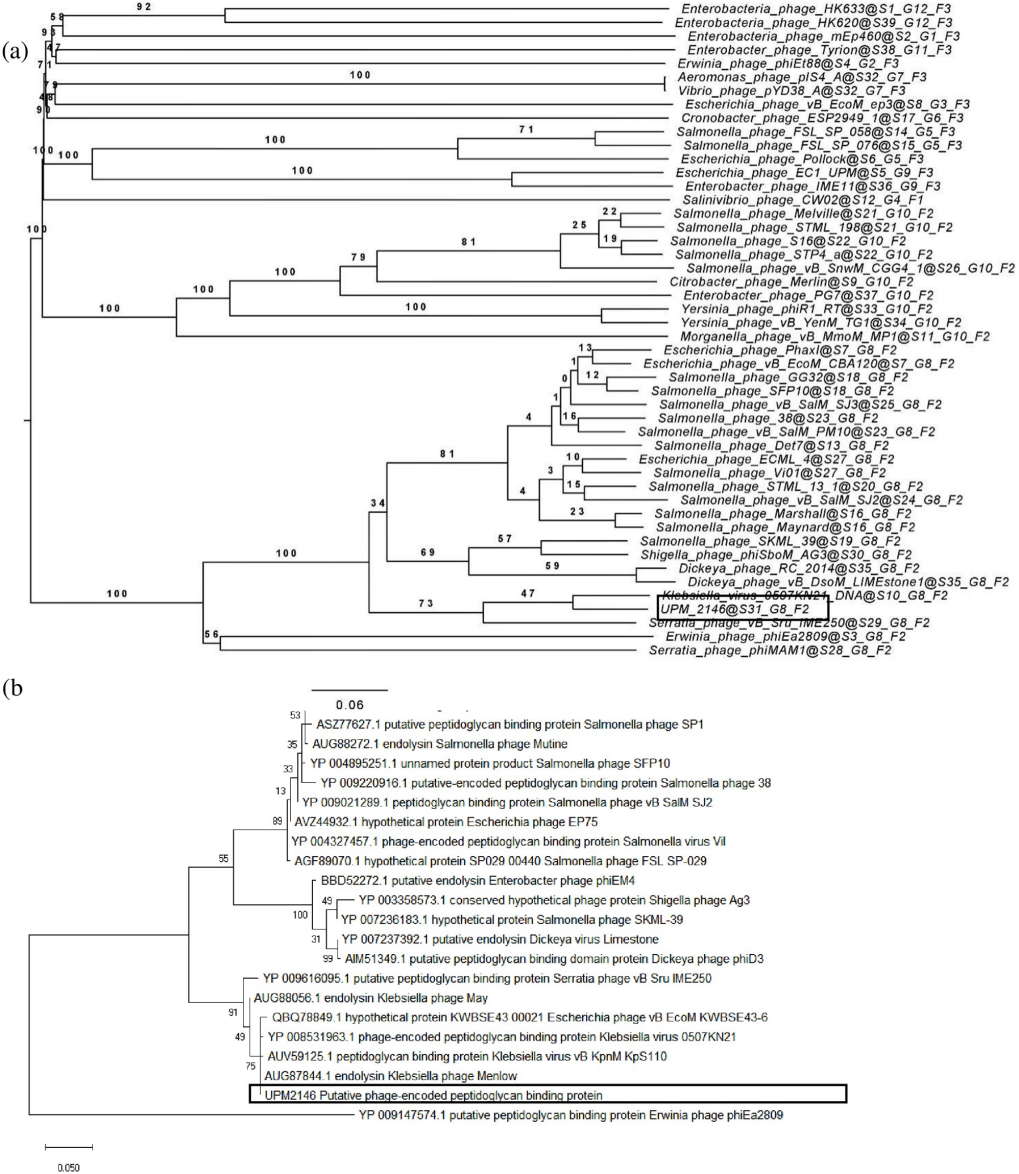
a. Phylogenic tree of UPM2146 show that the phylogenomic GBDP trees inferred using D6 formula and yielding average support of 58%, The numbers above branches are GBDP pseudo-bootstrap support values from 100 replications. The branch lengths of the resulting VICTOR tree are scaled in terms of D6 formula used. b. The evolution of UPM2146 of putative phage-encoded peptidoglycan binding protein had been drawn using phylogenic tree analysis using 1000 bootstrap, using the maximum likelihood (ML) method based on the Jones-Taylor-Thornton (JTT) model, the tree with the highest log likelihood (-1805.44) is shown. The percentage of trees in which the associated taxa clustered together is shown next to the branches. Initial tree(s) for the heuristic search were obtained by applying the Neighbor-Joining method to a matrix of pairwise distances estimated using a JTT model. The tree is drawn to scale, with branch lengths measured in the number of substitutions per site. This analysis involved 22 amino acid sequences. All positions containing gaps and missing data were eliminated (complete deletion option). There were a total of 261 positions in the final dataset. Evolutionary analyses were conducted in MEGA X [36].

UPM2146 was found to have a linear double-stranded DNA with a genome size of 160,795 bp and with a GC content of 46.31% (NCBI accession number MN478483). The genome contains 214 open reading frames (ORF) that can be transcribed to 81 proteins of known putative functions, 133 hypothetical proteins and 5 tRNAs as summarized in Fig 6. In addition, UPM2146 appear to have 89% similarity with *Klebsiella* virus 0507KN21 (Fig 7), suggesting that it may belong to *Caudovirales*, sub-family *Ackermannviridae* based on phylogenic tree analysis and using OPTSIL clustering yielded 39 (D6) species clusters, respectively. At the genus level, there were 12 (D6) clusters determined at the family level (Fig 8a). In addition, the presence of GP5 in the tail of the phage was predicted.

Furthermore, a phylogenic tree drawn by using Mega X software [36] based on UPM2146 putative phage-encoded peptidoglycan binding protein places UPM2146 within the newly proposed family of *Ackermannviridae* [43] in Fig 8b. UPM2146 was shown to be a lytic phage since it lacks integrase and toxin gene (PHASTER and PHACTS).

### Effectiveness of UPM2146 to kill *K*. *pneumoniae* 2146-infected zebrafish larvae

**Fig 9:**
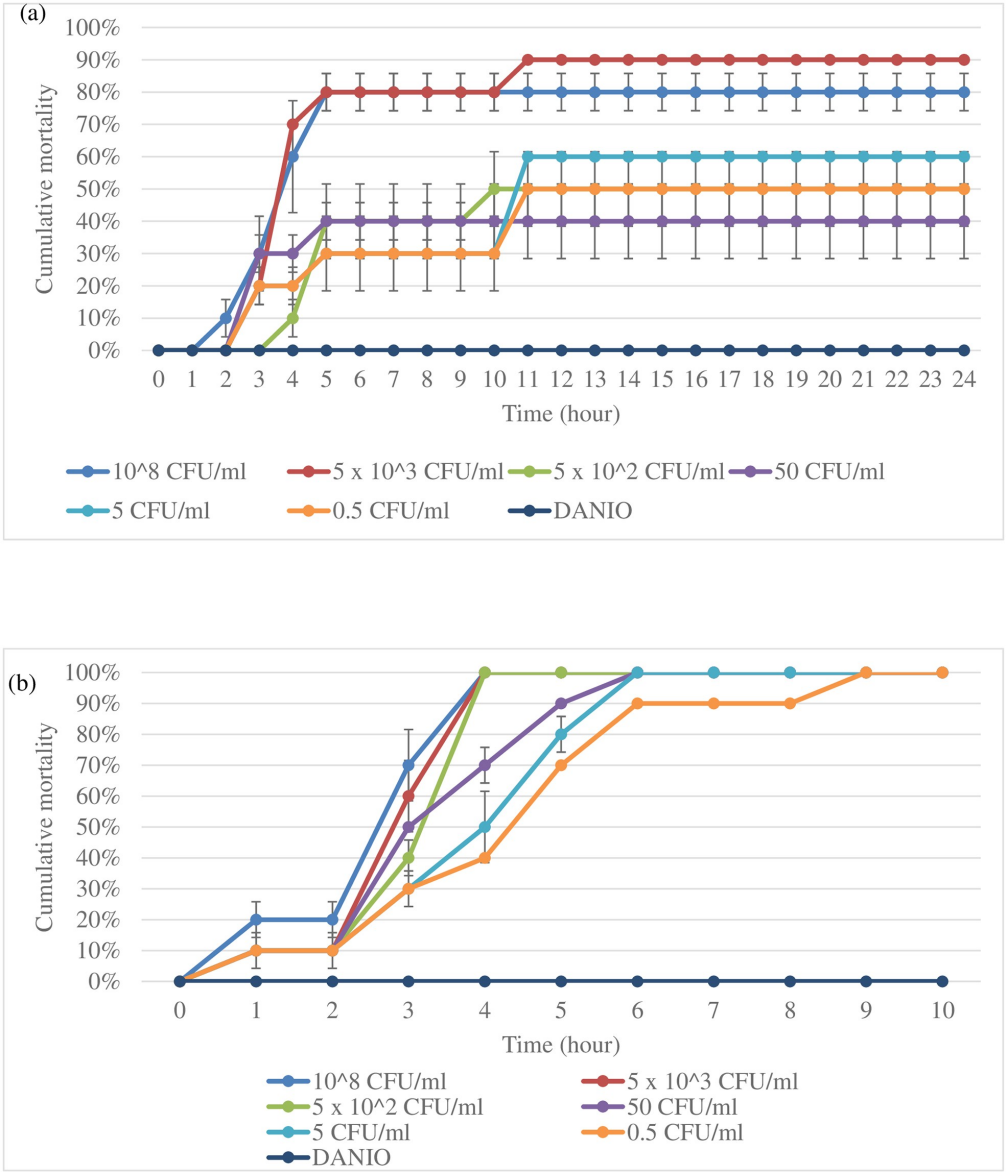
Survivability of zebrafish larvae based on morphological appearance. (a.) zebrafish larvae were able to survive up to 24 h when infected with *K*. *pneumoniae* 2146 for 30 min. while (b.) upon increasing the infection to 90 min the zebrafish larvae was able to survive up to 9 h. The experiment was done in triplicates.

**Fig 10:**
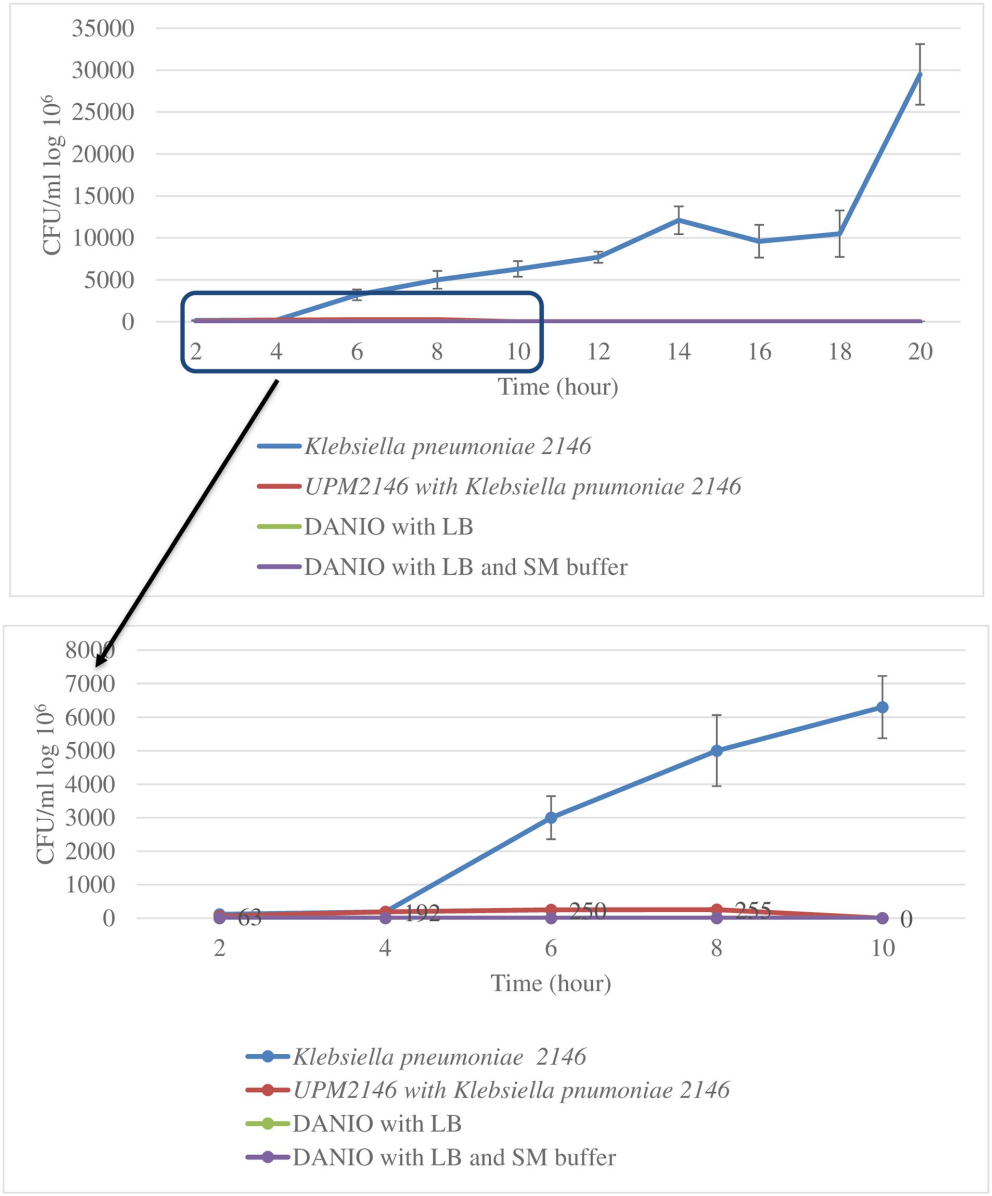
CFU count in zebrafish larvae. The phage UPM2146 managed to completely lyse *K*. *pneumoniae* 2146 at 10 h and remains zero until 20 h while the zebrafish larvae infected with bacteria (*K*. *pneumoniae* 2146) continue to increase till reaching 25.3 x 10^6^ CFU/ml. The experiment was done in triplicates.

The efficiency and safety of using UPM2146 in the treatment of *K*. *pneumoniae* infection *in vivo* were tested in a zebrafish larvae model. Based on morphological appearance, that is determined on whether the larvae exhibit hook shape tail which indicates that the larvae had died [44], the mortality rate was less, and the zebrafish larvae were able to survive up to 24 hours when infected for 30 min. On the other hand, the zebrafish larvae reached a mortality rate 100% at 10 hours when infected for 1 hour and thirty-minute; therefore, 30 min of infection was used for the CFU count experiment in zebrafish larvae (Fig 9a and 9b). The CFU count was conducted at an interval of 2 hours post-treatment with UPM2146 showing the presence of 1.17 x 10^5^ of colonies within zebrafish larvae in the group containing *K*. *pneumoniae* 2146 alone at the beginning and continuously increasing until reaching 2.53 × 10^7^ colonies compared to starting with 6.3 x 10^4^ colonies but reaching zero at 10 hours and remaining zero until 20 hours in the treated group (Fig 10).

## Discussion

In this study, UPM2146 was isolated from a polluted lake and was able to form halo-zones when plated on a *K*. *pneumoniae* lawn. This phenomenon was probably due to the release of soluble polysaccharide-degrading enzymes such as capsule depolymerase that was able to degrade the capsules of *K*. *pneumoniae* [45]. Interestingly, UPM2146 had shown to be a slight broad range but still limited to *Klebsiella* species as shown by host range and EOP. In addition, spot tests (for host range determination) may sometimes result in an overestimation of the capability of the phage to lyse the host bacteria due to other lysis effects not due to phage infection such as the effect of endolysin or bacteriocins from residual phage, bacteria or media components [19,46]. Categorizing host range is a challenge as the term “broad host range” has been used to describe phages which lyse multiple strains of the same species as in UPM2146 but also phages which lyse multiple species of bacteria. In the case where phages lyse multiple bacterial genera, the term “polyvalent” is used to describe the phage [46]. However, in reality, as shown with UPM2146, the phage while showing its ability to lyse several strains of *Klebsiella*, may only be efficient on one specific bacterial strain which is usually the host used to isolate the phage. This is due to the standard isolation procedure which involved enrichment with only one bacterial host. However, there are recent indications where using multiple hosts during isolation increases chances of isolating phages with broader host ranges [19]. In addition, the host range activity of bacteriophages can evolve and change with time [46–48].

UPM2146 has the requirements for therapeutic purposes since its optimum temperature and pH are 37°C and pH of 7 and it has the capability to lyse its host at a fast rate as shown by the one-step growth curve in Fig 3 [19]. Furthermore, UPM2146 was proven to be lytic based on turbidity assay due to its fast activity in lysing its host. This further confirms that *K*. *pneumoniae* 2146 has a low chance to develop resistance against UPM2146 [49] as well as any genes encoding integrase based on PHASTER [50]. The absence of integrase which is vital for the lysogenic life cycle further confirms that UPM2146 is a lytic phage. Moreover, this is supported by the prediction of being lytic based on PHACTS database (S1 and S2 Tables, S1 and S2 Figs) [31]. Furthermore, the presence of a putative phage-encoded peptidoglycan binding protein at ORF 19 shared 99.62% similarity with that belonging to *Klebsiella* virus 0507KN21 DNA [51]. Interestingly, UPM2146 belongs to the newly established *Ackermannviridae* family within the *Caudovirales* order based on the phylogenic tree analysis (Fig 8). UPM2146 is similar to *Klebsiella* virus 0507KN2 in which the pairwise distance between them was 0.0038 estimated using Poisson correction model (S2 Table). The genome size of UPM2146 which is over 155 kbp further supports that the phage belongs to *Ackermannviridae* [52] which is different from the other families (*Myoviridae*, *Siphoviridae*, and *Podoviridae*) within the *Caudovirales* order [53].

Moreover, UPM2146 is capable of encoding the muramidase endolysin which catalyzes the hydrolysis of the peptidoglycan in the *K*. *pneumoniae* cell wall, rupturing it at the end of the virulence cycle resulting in lysis from within [54]. In addition, the presence of the GP5 protein at the tail of the phage probably enables the mechanism of lysis from without leading to the breakdown of the bacterial cell wall [55]. Moreover, the genome seemed to encode for 133 hypothetical proteins with unknown functionality but they may have the potential to be used as new biologics after bioinformatics and proteomic analysis of hypothetical protein [56].

Turbidity assay (A_600nm_) of *K*. *pneumoniae* infected with UPM2146 showed that the phage was able to lyse the host bacteria and clear the turbidity within 1 hour at MOI of 0.02, 0.2 and 2. Thus, UPM2146 appears to have all of the requirements to be used for phage therapy [19,57] except for the ability to lyse a broad range of *K*. *pneumoniae* which can be overcome by using a phage cocktail [58]. In earlier studies, infected mice were used to prove the efficiency of phage in treating against *K*. *pneumoniae* [10–12]. However, in the present study, the zebrafish larvae model was used instead. It is important to note that as far as we know; testing the efficiency of phage using zebrafish larvae against *K*. *pneumoniae* has never been reported before. In a study by Marcoleta *et al*. [9], three types of *K*. *pneumoniae* were used to infect zebrafish larvae, but only one (*Kp* RYC492) was found to be highly virulent and caused 75% mortality after 72 hours post-infection (h.p.i.). In another study by Cheepurupalli *et al*. [14], clinical MDR *K*. *pneumoniae* at 10^7^ CFU/ml was used to infect the zebrafish resulting in 100% mortality at 24 h.p.i. In comparison with the current study, *K*. *pneumoniae* 2146 was extremely virulent to the zebrafish larvae; causing 100% mortality at 10 h.p.i. after 1 hour and 30 minutes infection at 10^3^ CFU/ml. However, 100% mortality of the zebrafish larvae was observed at 24 h.p.i. when the larvae was exposed to the bacteria for only 30 minutes. Furthermore, the c.f.u. count dropped to 0 at 10 hours onwards when UPM2146 was used to treat the infected zebrafish larvae at an MOI 0.3. This indicates that UPM2146 has the potential to be used for therapeutic purposes. On a side note, the zebrafish infection model is commonly used to test cytotoxicity [16] which in this study has proven UPM2146 is safe to be used.

## Conclusion

The continuous increase in drug-resistant bacteria had led scientists to search for alternative treatments to antibiotics such as phage therapy. In this study, UPM2146 had the ability to lyse multiple drug-resistant *K*. *pneumoniae* 2146 efficiently *in vitro* as well as four other *K*. *pneumoniae* strains with lesser efficacy. It had a short latent period, and its genome appears to be free from toxin and integrase genes. In addition, UPM2146 belongs to novel family (*Ackermannviridae*) within the *Caudovirales* order along with the presence of phage-encoded peptidoglycan binding protein (endolysin) that has the potential to be used for therapeutic purposes. UPM2146 was highly efficient in clearing the *K*. *pneumoniae* infection and can be considered as safe with the potential to be used as phage therapy against the bacteria although it is likely to be practically effective only when used in a phage cocktail due to its narrow host range.

## Supporting information

S1 Fig (PNG)

S2 Fig (PNG)

S1 Table (XLS)

S2 Table (XLSX)
