# Supplementary figures and images for: Klebsiella virus UPM2146 lyses multiple drug-resistant Klebsiella pneumoniae in vitro and in vivo
Source: PLoS One. 2021 Jan 8;16(1):e0245354. doi: 10.1371/journal.pone.0245354 (PMC7794032; doi:10.1371/journal.pone.0245354)

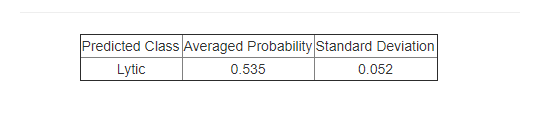

Supplement: S1 Fig — (PNG) [file pone.0245354.s001.png]

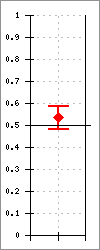

Supplement: S2 Fig — (PNG) [file pone.0245354.s002.png]
